# Evolution of major histocompatibility complex gene copy number

**DOI:** 10.1371/journal.pcbi.1007015

**Published:** 2019-05-16

**Authors:** Piotr Bentkowski, Jacek Radwan

**Affiliations:** Evolutionary Biology Group, Faculty of Biology, Adam Mickiewicz University in Poznań, Poland; National Institute for Public Health and the Environment, NETHERLANDS

## Abstract

MHC genes, which code for proteins responsible for presenting pathogen-derived antigens to the host immune system, show remarkable copy-number variation both between and within species. However, the evolutionary forces driving this variation are poorly understood. Here, we use computer simulations to investigate whether evolution of the number of MHC variants in the genome can be shaped by the number of pathogen species the host population encounters (pathogen richness). Our model assumed that while increasing a range of pathogens recognised, expressing additional MHC variants also incurs costs such as an increased risk of autoimmunity. We found that pathogen richness selected for high MHC copy number only when the costs were low. Furthermore, the shape of the association was modified by the rate of pathogen evolution, with faster pathogen mutation rates selecting for increased host MHC copy number, but only when pathogen richness was low to moderate. Thus, taking into account factors other than pathogen richness may help explain wide variation between vertebrate species in the number of MHC genes. Within population, variation in the number of unique MHC variants carried by individuals (INV) was observed under most parameter combinations, except at low pathogen richness. This variance gave rise to positive correlations between INV and host immunocompetence (proportion of pathogens recognised). However, within-population variation in host immunocompetence declined with pathogen richness. Thus, counterintuitively, pathogens can contribute more to genetic variance for host fitness in species exposed to fewer pathogen species, with consequences to predictions from “Hamilton-Zuk” theory of sexual selection.

## Author summary

Highly polymorphic genes of the Major Histocompatibility Complex (MHC) code for proteins responsible for presenting antigens to lymphocytes, thus initiating adaptive immune response. The polymorphism is driven by coevolution with parasites which are selected to evade recognition by MHC proteins. Expressing many MHC molecules could ensure that an individual could present antigens of most pathogen species encountered, but this comes at a cost, such as enhanced negative selection on lymphocytes leading to holes in T-cell receptor repertoire. Our simulations showed that evolution of the number of MHC genes in the genome is driven by a complex interaction between three factors we explored: pathogen richness, the intrinsic cost of expressing additional MHC variants, and pathogen mutation rate. In contrast to verbal arguments, our results indicate that pathogen richness does not always selects for MHC gene family expansion. Taking into account factors other than pathogen richness, in particular costs of expressing additional MHC variants which are still poorly understood, may help explain striking interspecific variation in the number of MHC genes. Counterintuitively, our results also demonstrated that opportunity for selection on immunocompetence should decrease with MHC gene family expansion.

## Introduction

Major histocompatibility complex (MHC) genes code for proteins that present pathogen-derived oligopeptides (antigens) to T-cells, thus initiating an adaptive immune response. MHC genes are highly polymorphic, with dozens to hundreds of variants typically segregating in natural populations (reviewed in [1–3]). This extreme polymorphism is thought to result from balancing selection imposed by pathogenic organisms [4, 5], and broadly-reported associations between MHC variants and susceptibility to infection are consistent with the role of pathogens in driving MHC evolution (reviewed in [3]). Correlative and comparative analyses reported positive associations between parasite community richness and the number of MHC alleles within a population and strength of positive selection on MHC [6–9], providing further support for the role of parasites in driving MHC diversity. However, a meta-analysis based on 112 mammalian species showed that the signs, let alone the strength, of such associations may vary between taxa [10]. Interpretation of these differences is hindered by the scarcity of theoretical work exploring the impact of parasite richness on MHC diversity.

The majority of MHC research has focused on amino acid sequence polymorphism. However, an aspect of MHC diversity that has received less attention is the number of MHC variants carried by individuals (in this article, we use the term “variants” to describe individual MHC diversity, which is the number of distinct MHC molecules carried by an individual; we prefer this to the term “alleles” often used in MHC literature, as the variants are not alleles in a strict sense, being often distributed over several, functionally equivalent MHC loci). This number of variants carried by individuals is typically much lower than the number found in the population. For example, in humans, there are 6–7 classical MHC loci, allowing for up to 12–14 different variants in a fully heterozygous individual, while the number of currently identified MHC alleles summed across those loci in the human population exceeds 17 000 (IPD-IMGT/HLA Database (8), Release 3.30.0). Given that most alleles segregating in a population are thought to be maintained by selection from pathogens [3], such discrepancy suggests that any individual’s MHC diversity is unlikely to be sufficient to efficiently respond to the whole spectrum of pathogens a host may encounter. This implies there is some intrinsic cost of expressing too high MHC diversity. One possible mechanism constraining evolution of individual MHC diversity is the deletion of self-reacting T-cells, during negative selection in the thymus. This deletion is likely to intensify with an increased number of expressed MHC variants, leading to a sub-optimal T-cell repertoire[11, 12, but see 13 for criticism]. Recently, this mechanism has been supported by the study of Migalska et al. [14], who reported a negative correlation between the number of expressed MHC class I variants and T-cell receptor repertoire in the bank vole. However, alternative mechanisms [reviewed in 13], such as increased risk of autoimmunity or the necessity to reach a critical concentration of MHC–peptide ligands at the surface of antigen-presenting cells, can also play a role.

However, there are huge differences among species in the number of MHC loci, ranging from a very few e.g. in chicken [15] or humans [16] to dozens in some rodents [17] or passerine birds [18]. This raises the question: why should stabilizing selection on individual MHC diversity lead to such different numbers of MHC loci in different species? Answering this question may have broad implications beyond immunogenetics and host-parasite coevolution. For example, it has been suggested that the exceptional evolutionary success of passerines, a family comprising ca. 70% of all bird species, has been facilitated by their supreme immunity due to extremely high numbers of MHC genes they harbour [19]. Furthermore, evolution of individual MHC diversity may have implications for biological conservation [20] or speciation [21].

Similarly to population-level polymorphism, interspecific differences in MHC copy number could be due to differences in the richness of parasites the species is exposed to, although studies which have examined this association are rare. O’Connor et al [22] found that among passerines, the number of unique MHC variants carried by an individual (which should correlate with the number expressed MHC loci) is lower in the Palearctic compared to Africa, which they ascribed to higher parasite species richness in the latter region. Similarly, Minias et al. [23] showed that passerine MHC expansion is related to migratory behaviour, likely in response to larger diversity of pathogens encountered by migratory species. In a more direct approach, Eizaguirre et al. [24] compared two lakes and two river populations of three-spined sticklebacks *Gasterosteus aculeatus* and found that lake populations, which systematically harboured more parasite species, had more MHC variants per individual. Similarly, Radwan et al. [25] found a positive relationship between a proxy for parasite load and individual number of MHC variants in ornate dragon lizard *Ctenophorus ornatus* populations inhabiting isolated patches of natural habitat. Interestingly, the authors did not find a significant association of parasite load with population-level allelic MHC richness and speculated that evolution of high copy number may weaken the balancing selection that might otherwise maintain high polymorphism. Similarly, Dearborn et al. [26] argued that high individual MHC diversity which arose in Leach’s storm-petrels, *Oceanodroma leucorhoa* by duplication followed by diversification of MHC class II genes should weaken advantage of heterozygosity at MHC. However, there is a lack of theoretical work on how parasite richness simultaneously affects MHC allelic richness and the number of MHC loci.

Here, we aim to fill this gap using computer simulations based on a framework that has previously been shown to be effective in recovering some of the most important features of MHC evolution, such as high polymorphism, frequency-dependent selection, heterozygote advantage and positive selection [27, 28]. The model simulates interactions of MHC molecules and antigens produced by pathogens by matching strings of bits, which can mutate both in hosts and in parasites [27, 28]. Here, we introduce a new feature to the framework to allow duplication and deletion of MHC genes. We then investigate how the number of pathogen species infecting a host affects the evolution of MHC allelic richness and the number of MHC loci.

## Results

**Fig 1:**
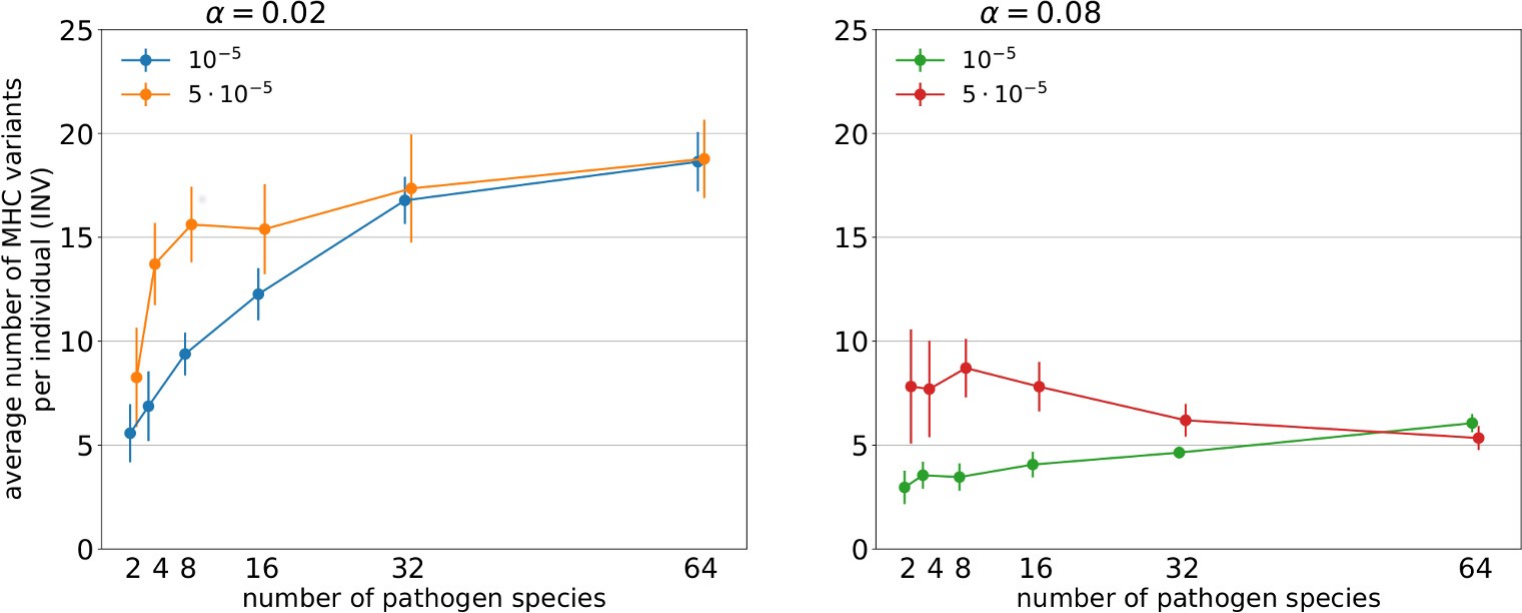
Relationship between the number of pathogen species and the average numbers of unique MHC variants present in a genome of a host under two penalty factors (α = 0.02; 0.08 –panels) and two pathogen mutation rates (μ_A_ = 10^−5^; 5 ∙ 10^−5^ –legends). The points represent the mean of the averaged values of simulations in a given parameter set with the 95% CI of the mean (bars).

Pathogen richness affected the number of unique MHC variants per individual (individual number of variants, INV henceforth) in a complex way, shaped by significant interactions with pathogen evolution rate and with the intrinsic cost of expressing more MHC variants (described by cost parameter α) (Table 1). Parasite species richness clearly increased INV at lower α, but at higher α there was little change in the INV across the levels of pathogen richness (Fig 1). There was also a significant pathogen richness × pathogen mutation rate interaction (Table 1), with the positive effect of higher pathogen mutation rate observed at low pathogen richness, but declining to zero as pathogen richness increased (Fig 1).

**Table 1:** Results of a generalized linear model analysing the effect of intrinsic cost α, pathogen mutation rate and pathogen richness on the average number of MHC variants per individual (INV) per simulation run.

	Estimate	SE	t-value	P-value
intercept	8.26	0.44	18.59	< 0.001
α	-3.99	0.63	6.25	< 0.001
mutation rate	-5.10	0.62	-8.12	< 0.001
pathogen richness	-0.04	0.01	-3.23	0.001
α × mutation rate	-0.13	0.90	-0.14	0.884
α × pathogen richness	0.17	0.02	7.51	< 0.001
m. rate × p. richness	0.09	0.02	4.49	< 0.001
α × m. rate × p. richness	0.005	0.03	0.17	0.860

**Fig 2:**
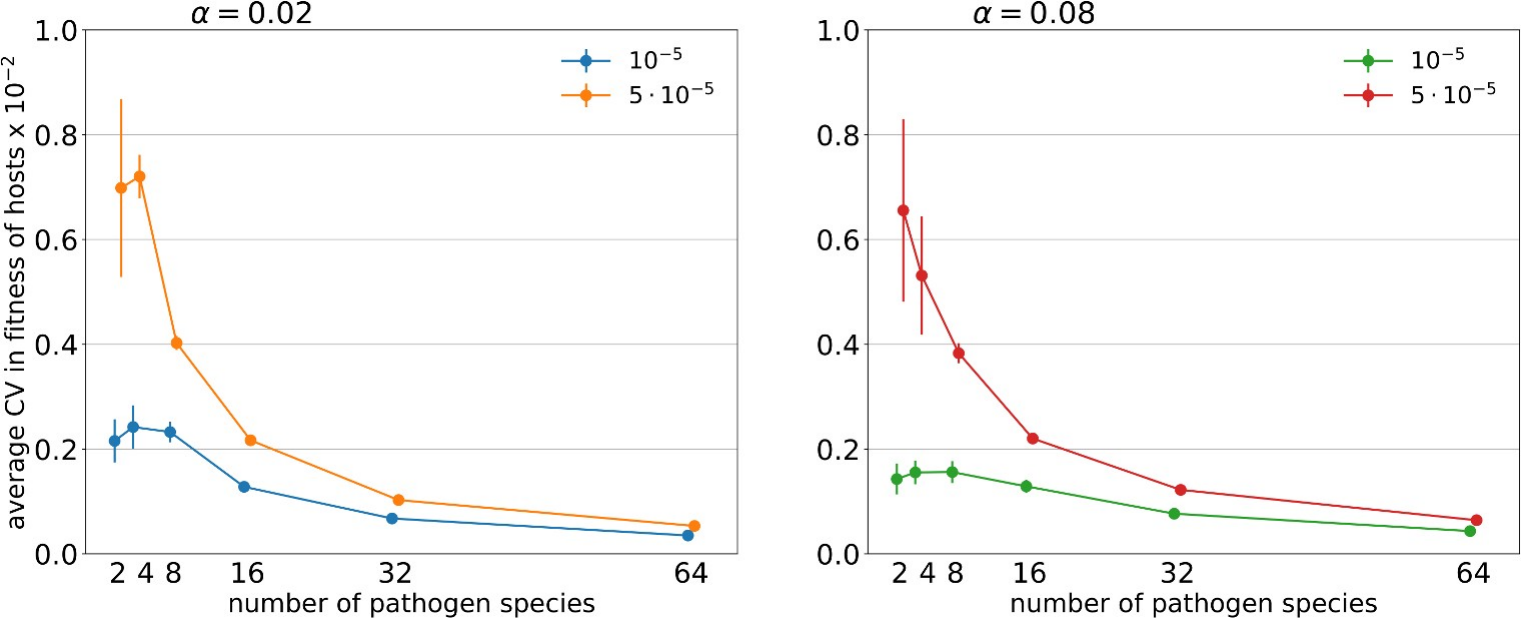
Relationship between the number of pathogen species and coefficient of variation (CV) in host fitness under two penalty factors (α = 0.02; 0.08) and two pathogen mutation rates (μ_A_ = 10^−5^; 5 ∙ 10^−5^). The average CV fitness is normalized for the number of pathogen species and the number of pathogen generations per one host generation. The points represent the mean of the averaged values of simulations in a given parameter set with the 95% CI of the mean (bars).

The selection acting on host MHC genotypes, as measured by coefficient of variation (CV) in host fitness (which in our simulation was determined solely by host immunocompetence, i.e. the proportion of pathogens recognized), was shaped by the significant interaction between pathogen richness and mutation rate (Table 2). CV in host fitness was much higher at higher pathogen mutation rate when pathogen species number was low (Fig 2). However, the differences between mutation rates declined, as did CV itself, with an increase in pathogen richness.

**Table 2:** Results of a general linear model analysing the effect of intrinsic cost α, pathogen mutation rate and pathogen richness on the average coefficient of variation in host fitness per simulation run.

	Estimate	SE	t-value	P-value
intercept	0.0016	0.0003	4.43	<0.001
α	-0.0014	0.0061	-1.85	0.064
mutation rate	92.68	10.04	9.22	< 0.001
pathogen richness	2.38e^-5^	1.44e^-5^	-1.64	0.100
α × mutation rate	-70.49	170	-0.41	0.678
α × pathogen richness	2.63e^-4^	2.26e^-4^	1.16	0.245
m. rate × p. richness	-2.06	0.40	-5.13	<0.001
α × m. rate × p. richness	5.56	6.29	0.88	0.377

**Fig 3:**
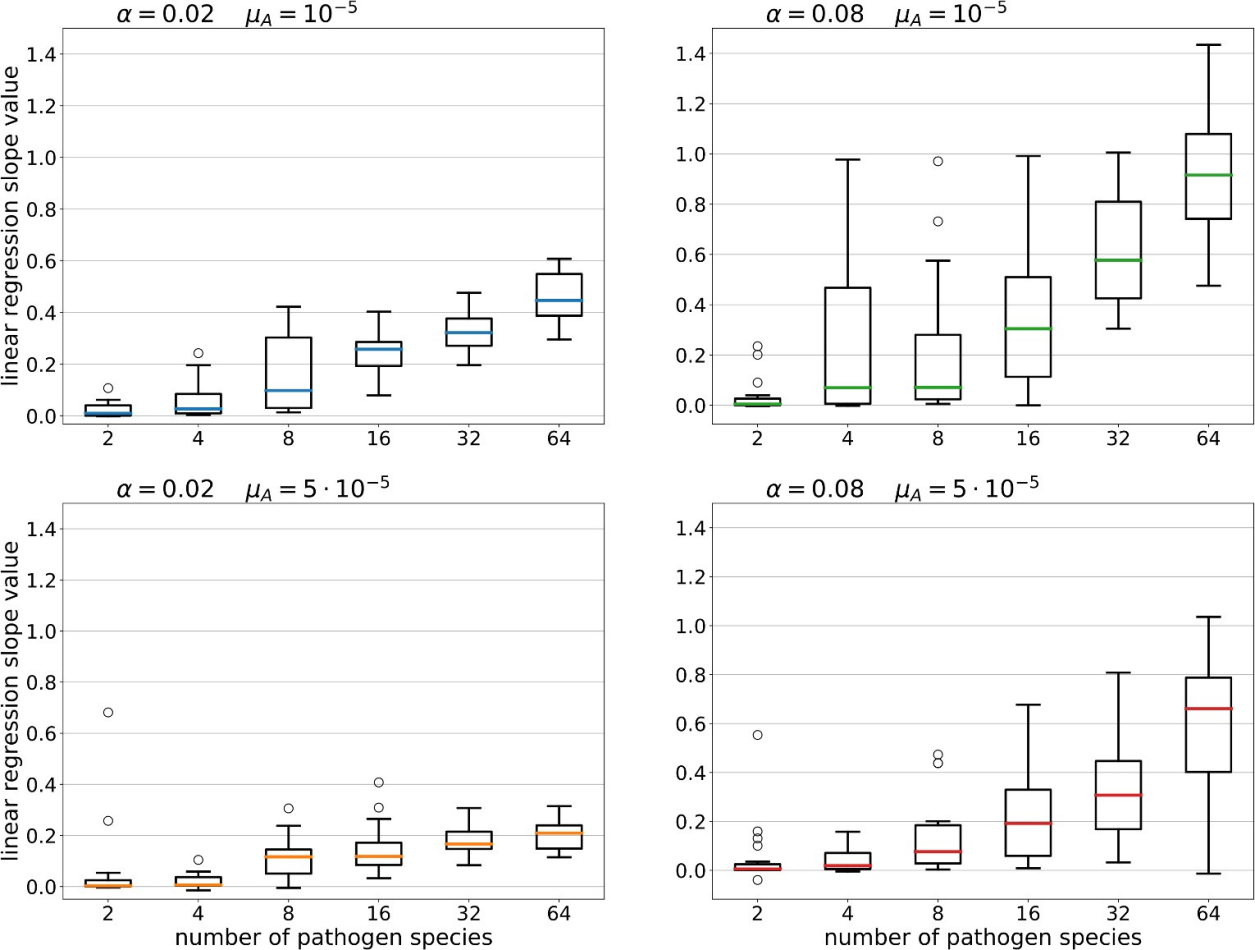
Coefficients of regression of INV on pathogen presentation ability for various combinations of parameters. For each simulation run we calculated the linear regression between the number of unique MHCs in individuals and the number of infections they were able to present to the immune system. Boxplots (median and quartiles) summarize the slopes of regression for each parametrization.

We observed considerable within-population variation in the INV under most scenarios, except when the number of pathogens was very low (S4 Fig). The slopes of the relationship between the number of pathogens presented to the immune system and INV increased with pathogen richness, but slopes were generally low at higher pathogen mutation rate (Fig 3).

**Fig 4:**
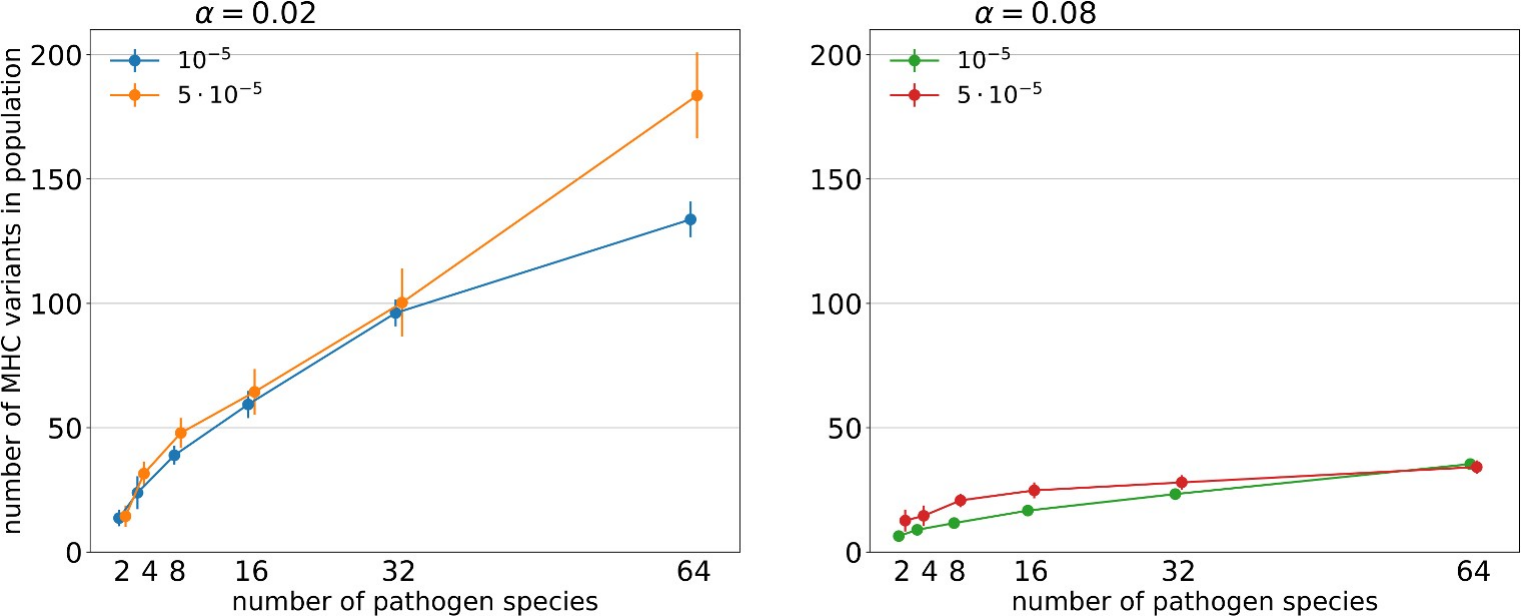
Relationship between the number of pathogen species and the average numbers of unique MHC variants present in a population under two penalty factors (α = 0.02; 0.08 –panels) and two pathogen mutation rates (μ_A_ = 10^−5^; 5 ∙ 10^−5^ –legends). The points represent the mean of the averaged values of simulations in a given parameter set with the 95% CI of the mean (bars).

The number of MHC variants segregating in a host population (PNV henceforth) was driven by the significant three-way interaction between α, pathogen richness and mutation rate (Table 3). PNV generally increased with pathogen richness (Fig 4, Table 3), but the increase was lower at α = 0.08. High pathogen mutation rate increased PNV only at the combination of low α and high parasite richness (Fig 4). Interestingly, at low α, PNV largely reflected INV, whereas at high α PNV increased (Fig 4) despite that INV did not (Fig 1; see S5 Fig for correlation between INV and PNV).

**Table 3:** Results of a generalized linear model analysing the effect of intrinsic cost α, pathogen mutation rate and pathogen richness on the average number of MHC variants in a population (PNV) per simulation run.

	Estimate	SE	t-value	P-value
intercept	15.82	1.53	10.30	< 0.001
α	-3.723	2.20	1.68	0.091
mutation rate	-8.21	2.17	-3.78	<0.001
pathogen richness	0.31	0.05	6.25	<0.001
α × mutation rate	8.60	3.11	2.76	0.006
α × pathogen richness	2.26	0.08	27.74	< 0.001
m. rate × p. richness	0.13	0.07	1.84	0.066
α × m. rate × p. richness	-0.70	0.11	-6.11	<0.001

## Discussion

Our model showed that under the Red Queen-like dynamics of MHC evolution, evolution of INV is shaped by a complex interaction of several factors, including pathogen richness, pathogen mutation rate, and the intrinsic cost of expressing many MHC molecules. Verbal arguments [e.g. 8, 22, 23] assumed that INV should generally increase with the number of pathogen species. In our simulations, this was the case only under some parameter combinations, and the form of the relationship depended both on the intrinsic costs of expressing additional MHC variants and on pathogen mutation rate. INV consistently increased across the investigated range of parasite species when the intrinsic cost of large MHC repertoire was small. However, with higher values for the cost factor (α), we did not observe such an increase. This shows that high pathogen richness will not necessarily lead to the evolutionary expansion of MHC gene family. Little is known about the nature of the intrinsic costs of MHC expansion, and even less on how taxa differ in this respect, and therefore we have not modelled any particular mechanism underlying these costs in our simulations. The prevalent explanation is that high MHC diversity increases negative selection of self-reactive T-cell receptors [11, 12], impairing efficiency of immune response. This scenario has recently been supported in bank voles, where TCR repertoire has been demonstrated to decrease with the number of MHC class II variants [14]. Under such a scenario, intermediate numbers of MHC variants should result in the most efficient clearing of infections, as has been observed in some empirical studies, including bank voles [29–31]. However, several studies utilising extensive variation in INV present in passerine birds have observed either no such a relationship, or negative associations between INV and infection [e.g. 32, 33–35]. This suggests that the nature of the intrinsic costs of expressing many MHC variants may differ between passerines and mammals. One possibility is that expressing too many MHC variants does not compromise passerine TCR repertoire in a way similar to that observed in bank voles [14], allowing rapid expansion of MHC gene family (compare Fig 1). The study of TCR repertoires in birds, and the way they are shaped by MHC, emerges as an attractive target for future studies.

More generally, understanding inter-specific difference in INV will require extensive study of intrinsic costs of expressing additional MHC variants across vertebrate taxa. Our model indicates that higher pathogen richness is unlikely to explain a spectacular expansions of MHC gene family, such as those observed among passerines. Ancestrally, birds have been characterised by a small number of MHC genes, which is still observed in non-passerines [23]. Our results suggest that expansion to dozens of MHC loci observed among some passerine superfamilies (Sylvioidea, Passeroidea and Muscicapoidea [23]) would require the number of pathogen in these lineages to be manifold higher compared to basal groups (compare Fig 1), which does not appear biologically feasible.

Another factor which influenced the evolution of INV in our simulations was pathogen mutation rate, the effect of which was most pronounced at low pathogen species numbers (Fig 1). This pattern was mirrored by variance in host fitness (measured as CV), which was the highest for high pathogen mutation rate combined with low pathogen richness (Fig 2). Host haplotypes with more MHC variants should be more likely to carry a variant conferring resistance to a parasite, but efficient evasion of MHC-recognition by fast-evolving pathogens could weaken association between INV and pathogen recognition, consistent with our results (Fig 3). Still, efficient parasite evasion should favour novel MHC variants [28], and such variants are more likely to arise when the number of copies in the genome is high. When average number of MHC variants is already high, however, possessing an extra MCH copy provides relatively smaller advantage in terms of potential for beneficial mutation. This may explain why the effect of pathogen evolution rate on INV was observed only at low pathogen richness (where INV was relatively low).

Similarly, high CV in host fitness at low numbers of pathogen species likely results from the fact that a haplotype that is resistant to a prevalent pathogen genotype (of any species) will gain considerable advantage, whereas with many pathogen species resistance to any given pathogen contributes relatively less to fitness. This may explain why CV in host fitness declined with pathogen richness, which may have interesting implications for predictions stemming from Hamilton and Zuk’s (1982) theory of sexual selection. This theory poses that costly epigamic traits, such as long feathers or bright colouration, are subject to mating preferences because they reflect the genetic aspect of resistance to pathogens. At the interspecific level, the Hamilton-Zuk hypothesis predicts that higher risk of parasite infection should enhance sexual selection for extreme values of such epigamic traits, because of increasing contribution of pathogens to genetic variance in fitness (Hamilton and Zuk 1982, Berlanger and Zuk 2014). Paradoxically, our results indicate that while host genetic diversity for resistance (measured by the number of MHC variants segregating in populations) increased, the variance in host fitness decreased. Our results thus indicate that if the number of pathogen species attacking the host is used as a measure of selective pressure from pathogens, the predicted relationship with an elaboration of epigamic traits might be counter-intuitive.

INV was positively correlated with pathogen recognition ability (Fig 3), as assumed by models of copy-number evolution [11, 12]. Nevertheless, our simulations suggest no such association should be expected when the number of MHC variants in the species is low (Fig 3). Indeed, in root voles *Microtus oeconomus* and guppy fish *Poecilia reticulata*, both characterised by a low to a moderate number of MHC loci (1–3), possessing particular variants has been shown to be more important than the number of expressed MHC loci [36, 37].

More interestingly, INV was not a good predictor of pathogen recognition efficiency when parasites evolved fast (Fig 3). As discussed above, fast-evolving parasites are more effective in evading recognition by MHC haplotypes prevalent in a population than slow-evolving ones. In consequence, when parasites evolve fast, possessing a rare-but-resistant MHC variant should have more of an effect on resistance than possessing many variants.

Our simulations revealed tight associations between PNV and INV, but the slope of the associations depends on the intrinsic cost of expressing additional variants (S5 Fig). At high α, where increase in pathogen richness does not result in a consistent increase in INV, PNV nevertheless increases, resulting in slope >1. At low α, at which INV is more free to evolve, PNV largely reflects INV, which implies that when selection from many parasites favours gene duplication, per-locus polymorphism may change very little. Our results may explain the findings of comparative analyses showing that high pathogen richness is sometimes not found to be associated with MHC allelic richness (a per-locus measure of variation), despite its effect on the rate of molecular evolution at MHC antigen binding sites [8, 9]. Two recent comparative studies [22, 23] demonstrated that among passerines, individual number of MHC variants decreases with such likely correlates of pathogen richness as latitude or migratory behaviour (although we know of no work directly linking INV to parasite richness). It would be interesting to see if INV could explain PNV in this system, as predicted by our model.

Concluding, our study showed that in general, pathogen richness selects for expansion of MHC gene family, but is unlikely to explain striking inter-specific differences in the number of MHC genes. The latter can be can be modulated both by the rate at which parasites evolve and, probably more critically, on the strength of mechanisms selecting against the high number of copies in the genome. These mechanisms are not well understood, but warrant investigation as potential causal factors underlying differences in MHC genes family sizes between species. In species which evolved high INV under selective pressure from many pathogen species, within population variation in INV can nevertheless be maintained. Despite high variation in INV, host variance in immunocompetence should, according to our model results, be lower in species experiencing selection pressure from higher diversity of parasites.

## Methods

### Simulation outline

The model is based on an approach first used by Borghans et al. [27], which simulated interactions between the peptide-binding grooves of MHC molecules and antigens derived from pathogens by aligning two strings of zeros and ones (bitstrings). In our model, each MHC molecule was represented as a 16-bit-long string, which can be thought of as a representation of the amino acids that form pockets implicated in the specificity of antigen binding (there are 12–23 polymorphic sites contacting antigens in human MHC molecules [38]). A pathogen was represented by a single 6000-bit long antigenic molecule, which was tested for a match with host MHC at all possible 16-bit epitopes which could be produced from the antigenic molecule. Antigen binding occurred when there was a match in all position of the bit strings representing the peptide bindig groove of MHC molecules and an epitope (S1 Fig). Utilising 16 bits, we could simulate 65,536 (2^16^) MHC epitopes. The probability of finding a random 16-bit sub-string (epitope) in a random 6000 bit antigen was approximately 0.084, a number corresponding to the empirical estimates of an MHC molecule binding a random epitope produced by viral pathogens [39, 40]. The way we simulated antigens differed from that in Borghans et al. [27] and earlier adaptations of their approach [e.g. 28, 41] in which a single parasite was represented by a set of 20 independent, 16-bit-long antigens, and 7 matched bits were used as a threshold for pathogen recognition. The rationale for simulating a long antigenic molecule and a higher threshold number of matching bits was that it reduced the number of recognition motifs shared between pathogen species, and, additionally, it facilitated further diversification of species-specific motifs by conserving some of them in a species-specific manner (see below). Nevertheless, the probability of binding a random antigen produced by a given pathogen remained broadly consistent with those earlier studies [27, 28, 41].

Hosts co-evolved with a variable number (2–64) of haploid pathogen species, which, to simplify simulations, had population sizes equal to that of their hosts [as in previous studies, e.g. 28]. Instead of simulating larger pathogen populations (as would have been observed in nature), higher probability of a mutation in large populations was emulated by a higher pathogen mutation rate. There were 10 pathogen generations per one host generation to reflect the fact that pathogens typically have faster generation times than hosts.

The fitness of pathogen haplotypes was proportional to the number of hosts a pathogen successfully infected, and host fitness was proportional to the number of pathogens recognized (see below for details). The next generation of both hosts and pathogens was drawn in proportion to their fitness. The algorithm described above effectively simulates a host-parasite co-evolution system with Red Queen dynamics [see 28 for more details].

### Hosts

MHC genes (i.e. 16 bit-long strings) were located on one diploid pair of host chromosomes. The size of the host population was fixed at 1000 individuals. These individuals were exposed to one, randomly chosen individual of each pathogen species. If the infection was successful (i.e. the pathogen was not recognized by any of the host’s MHC genes), the parasite clone could evolve in the host for 10 generations, ecologically excluding infections by other clones of the same species. If the infection was unsuccessful, a new, randomly selected individual attempted an infection in the next pathogen generation; if successful, this pathogen would be allowed to reproduce until 10 pathogen generations were completed. After 10 pathogen generation passed, host fitness was determined. The fitness was proportional to the number of pathogens presented by the host, but we additionally introduced a cost of having additional MHC variants (see below). The cost was introduced to reflect various mechanisms thought to counteract unconstrained expansion of MHC region [11, 13]. Our preliminary analyses indicated that the number of MHC loci rapidly increased and did not stabilise even after thousands of generations if no cost was introduced. The host fitness function was calculated according to the equation:

$$f_{host}=P\cdot e^{-{\left(\alpha N\right)}^{2}}$$ (1)

where *P* is the number of pathogen species a host recognized (thus avoiding infection), *N* is the number of unique MHC variants in the host’s genome and *α* is the cost factor. The cost factor *α* was selected to achieve a realistic number of unique MHC types in an individual (i.e. from a few to few dozens).

After interactions with pathogens (across 10 pathogen generation cycles), hosts reproduced with probability proportional to their fitness. We have not modelled separate sexes (i.e. our hosts were equivalent to out-crossing hermaphrodites). During reproduction, each of the diploid mates provided one chromosome (selected randomly) to the resulting progeny. Each mating resulted in one offspring, but individuals could be selected for mating more than once (which was more likely for high fitness hosts), and random mating pair selection was repeated until the size of the host population *N*_*H*_ was restored.

Host chromosomes could undergo two types of mutations: micromutations within the 16-bit string, and copy number mutations. Micromutations were represented by a flip of a single bit with a given probability. This can be thought of as a non-synonymous substitution in an antigen binding site of MHC molecule, which could occur as a non-synonymous mutation, or micro-recombination (the latter may be the predominant mode of mutation at human MHC [42]). For the sake of consistency with previous simulation studies, in which mutation rates were given as the probability of change in MHC molecule as a whole (replacement of old MHC with a new one), we report the mutation rate per MHC molecule (i.e. 16 bit string), which translates into per bit rate according to the equation:

$$\mu_{bit}=1-{\left(1-\mu_{MHC}\right)}^{1/16}$$ (2)

where *μ*_*MHC*_ is the mutation rate per MHC peptide, *μ*_*bit*_ is the mutation rate per single bit in the MHC PBR (see also S1 Appendix in [28]. We used a host mutation rate of 10^−4^ per MHC molecule (or 6.25 × 10^−6^ per PBR), which appears realistic based on published literature [42]. We also simulated “macromutations” in MHC, which could be thought of as recombination or gene conversion of large fragments of an exon coding for peptide binding groove. Following earlier work [27], we simulated macromutations by producing random strings of bits. However, mutation mode have not qualitatively affect our results (S6 Fig), therefore in the main text we only present results for micromuations.

Copy number of MHC genes could change via duplication or deletion. Duplication was modelled by adding a new copy of the original sequence on the same chromosome, and during deletion, a gene disappeared from the chromosome. However, the algorithm did not allow the number of MHC loci to go below 1 per chromosome. Each gene could be duplicated with probability 10^−3^ and deleted with probability 10^−3^, which is higher than direct empirical estimates for large structural variant indels in human genomes [43], but was the minimum necessary for the number of copies to stabilise within realistic computing time. Neo- or sub- functionalization of duplicated loci could occur by mutations described above.

### Pathogens

We simulated a variable number of haploid pathogen species, with the population size of each species equal to that of the host. A species was initiated as a single antigen, and thus individuals were sharing the evolutionary origin and history within species, but separate species were initialized independently. Because the possible number of distinct 6000 bit antigens is very large (~1.5 × 10^1806^), pathogen species showed little overlap in their antigenic profiles (S2 Fig; the probability that a random 16-bit-long sub-string will be present in both of two random and independent 6000-bit-long strings equals to ~0.084^2^). We trialled a variant of the simulations in which each pathogen species had a randomly-assigned, species-specific 33% of bits conserved, but this did not result in a different interpretation, and we do not report results from this version. Pathogen haplotypes were selected for reproduction with probability proportional to the number of hosts they had infected. During each of 10 pathogen generations, every host was matched with a randomly selected individual of each pathogen species and the outcome of the infection was evaluated according to the bit-matching rules described above. A pathogen species could infect an individual host only once per host generation. The successful pathogens reproduced parthenogenetically by producing 'clonal' progeny. The progeny could mutate by changing a single bit to the opposite before advancing to the next round of infections. To examine the role of pathogen evolution rate on our results, we simulated two pathogen mutation rates: 10^−5^ and 5 × 10^−5^_._ These values resulted in the host-parasite coevolution we sought to produce in our simulations. Exploratory analysis showed that at lower pathogen mutation rates than reported above, pathogens were unable to adapt to host genotypes fast enough, whereas at higher mutation rate fitness differences between host genotypes were small, precluding effective co-evolution [see 44]. For comparison, the influenza virus NS gene mutates at a rate of 1.5 × 10^−5^ [45].

### Implementation

The model’s program was written in C++14 language, which generates a number of text files of simulation results that were then analysed and plotted using Python scripts. The general scheme of the algorithm is shown in S3 Fig. The source code and its documentation can be obtained from https://github.com/pbentkowski/MHC_Evolution. Summaries of the model’s parameters and their values are given in Table 4. Each combination of parameters was run 20 times, except for the most computationally demanding simulations with 64 pathogens, which were run 10 times.

**Table 4:** Parameters of the model.

Parameter description	Symbol	Values
Host population size (number of individuals)	*N*_*H*_	1000
Pathogen population size of a species (number of individuals) ^1^	*N*_*P*_	1000
Number of pathogen species in the simulation	*S*	1, 2, 4, 8, 16, 32, 64
Antigen length (number of bits)	*a*	6000
MHC’s protein-binding region length (number of bits)	*m*	16
Number of pathogen generations per one hosts generation	*K*	10
Total number of hosts generation (a.k.a. simulation time)	*G*	5000
Probability of mutation in antigen (per site) ^2^^,^^3^	*μ*_*A*_	10^−5^, 5 · 10^−5^
Probability of mutation in MHC PBR (per protein) ^2^^,^^4^	*μ*_*MHC*_	10^−4^
Probability of deletion of MHC gene ^2^	*μ*_*del*_	10^−3^
Probability of duplication of MHC gene ^2^	*μ*_*dupl*_	10^−3^
Cost factor for the host selection function	*α*	0.02, 0.08

^1^ total number of pathogen individuals was equal to N_p_· S

^2^ probability per reproduction event

^3^ probability per site (flip of a single bit)

^4^ probability of change of the whole MHC (change in any given site)

### Statistical analysis

For evaluation purposes, we considered the last 1250 host generation when the dynamics of the host-parasite co-evolution stabilised in term of the numbers of MHC variants in both populations and individuals. For that period and for each run, we calculated mean PNV and mean CV in host fitness (pathogen presentation ability) by averaging it over 1250 latest host generations. To calculate mean INV, we first averaged across individuals at a given time step, and then took the averaged simulated values across 1250 latest host generations. Coefficients of regression of INV on pathogen presentation ability (Fig 3) was based on a population 'snapshot' at host generation #5000 (the last one), when we recorded detailed information on each host (what genes they had, what pathogen species they presented). These data are available in Supplementary File 1. Results were analysed with linear models, with an average INV, PNV or CV in host fitness as a response variable, and α, pathogen mutation rate and pathogen richness, and their interactions, as fixed factors. Statistical analyses were done in R 3.4.2.[46].

## Supporting information

S1 Fig The schema of pathogen presentation by a single MHC protein binding region (PBR). A bit string representing MHC PBR (16-bits-long) slides along the bit string representing antigen (6000-bits-long) until it will encounter an identical sub-string (epitope) in the antigen what leads to the presentation of the pathogen. If all of the host’s MHCs will reach the end of the antigen without finding a matching sub-string, the host gets infected with this pathogen species.
(TIF)

S2 Fig The schema of the simulation flow. The inner loop represents 10 pathogen generations during one host generation (the outer loop).
(TIF)

S3 Fig Similarities of antigen bit-strings measured between pathogen species (at the start and the end of simulations–double plot panels) and within three randomly selected species (at the end of the simulations). Presented are 4 runs with 64 pathogen species under two penalty parameters (α = 0.02; 0.08) and two pathogen mutation rates (μ A = 10^–5^; 5 ∙ 10^–5^). We used Hamming distance, a measure of similarity where two bit-strings of length n will have 1⁄2 n similar bits if they were generated randomly. At initialisation, a species will consist of a single copy of the same antigen, but species differ from each other.
(TIF)

S4 Fig Examples of distribution of numbers of unique MHC alleles in individuals. In columns are the two penalty parameters α = 0.02; 0.08 and two pathogen mutation rates μA = 10^–5^; 5 ∙ 10^–5^ (see descriptions above the figure). Rows contain runs with the same number of pathogen species (bold numbers on the right). Each panel represents one run that had its mean number of unique MHCs most similar to the mean calculated from all simulation of the same parametrization.
(TIF)

S5 Fig Correlation between the mean INV and mean PNV in each simulation run under two penalty parameters (α = 0.02; 0.08). Note, the y-axes have different scales. On the legend dots indicate runs with pathogen mutation rates μA = 10^–5^, triangles μA = 5 ∙ 10–5. Colours correspond to the number of pathogen species in the simulation (see the legend beneath the panels).
(TIF)

S6 Fig Comparison of simulation results with host macromutations (colour lines) to those with micromutations (grey lines, same as on Fig 1). The points represent the mean of all the averaged values of simulations in a given parameter set with the 95% CI of the mean (bars).
(TIF)

S1 File Excel sheet containing simulation results. (XLSX)
