# Supplementary figures and images for: Evolution of major histocompatibility complex gene copy number
Source: PLoS Comput Biol. 2019 May 16;15(5):e1007015. doi: 10.1371/journal.pcbi.1007015 (PMC6541284; doi:10.1371/journal.pcbi.1007015)

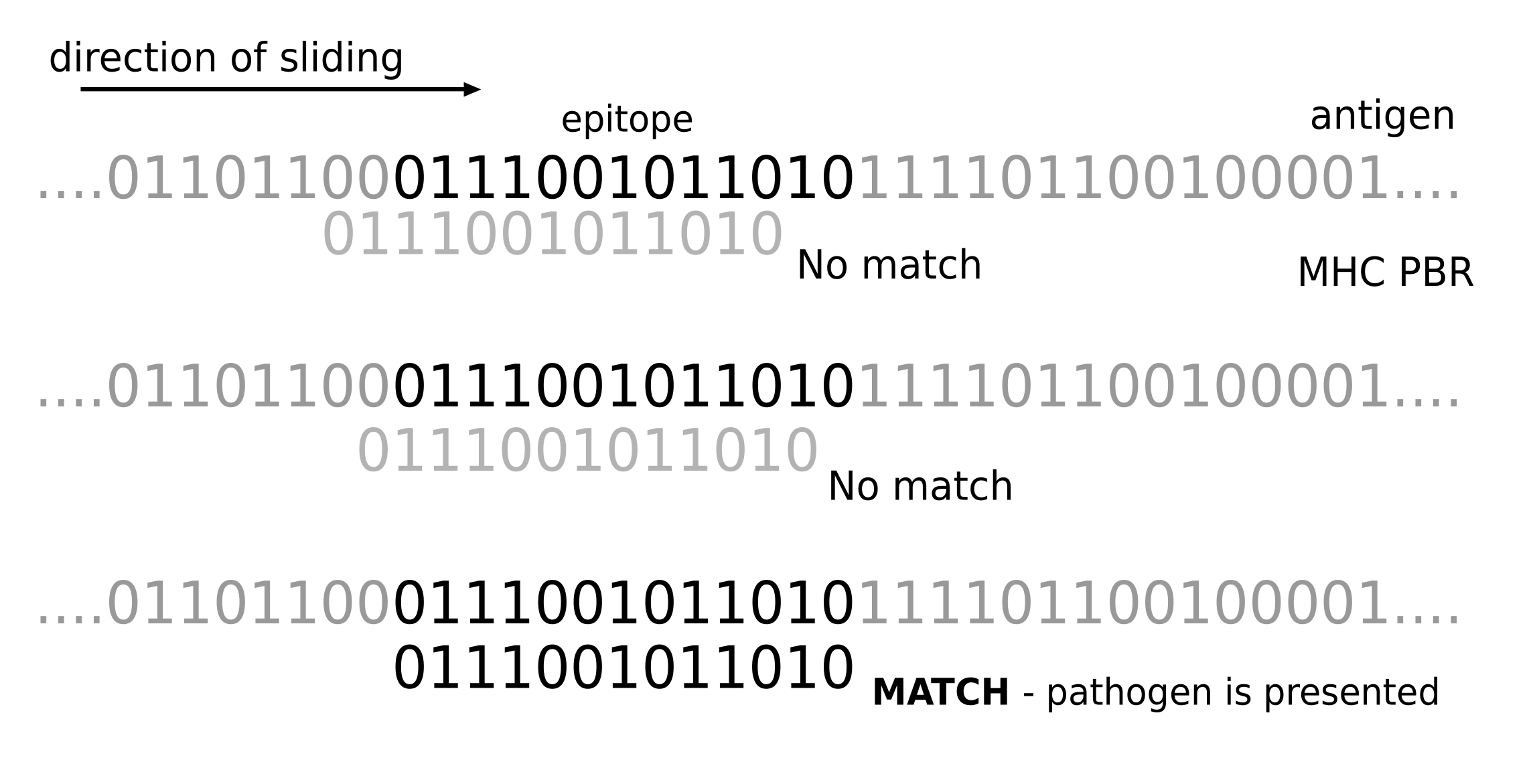

Supplement: S1 Fig — A bit string representing MHC PBR (16-bits-long) slides along the bit string representing antigen (6000-bits-long) until it will encounter an identical sub-string (epitope) in the antigen what leads to the presentation of the pathogen. If all of the host’s MHCs will reach the end of the antigen without finding a matching sub-string, the host gets infected with this pathogen species. (TIF) [file pcbi.1007015.s001.tif]

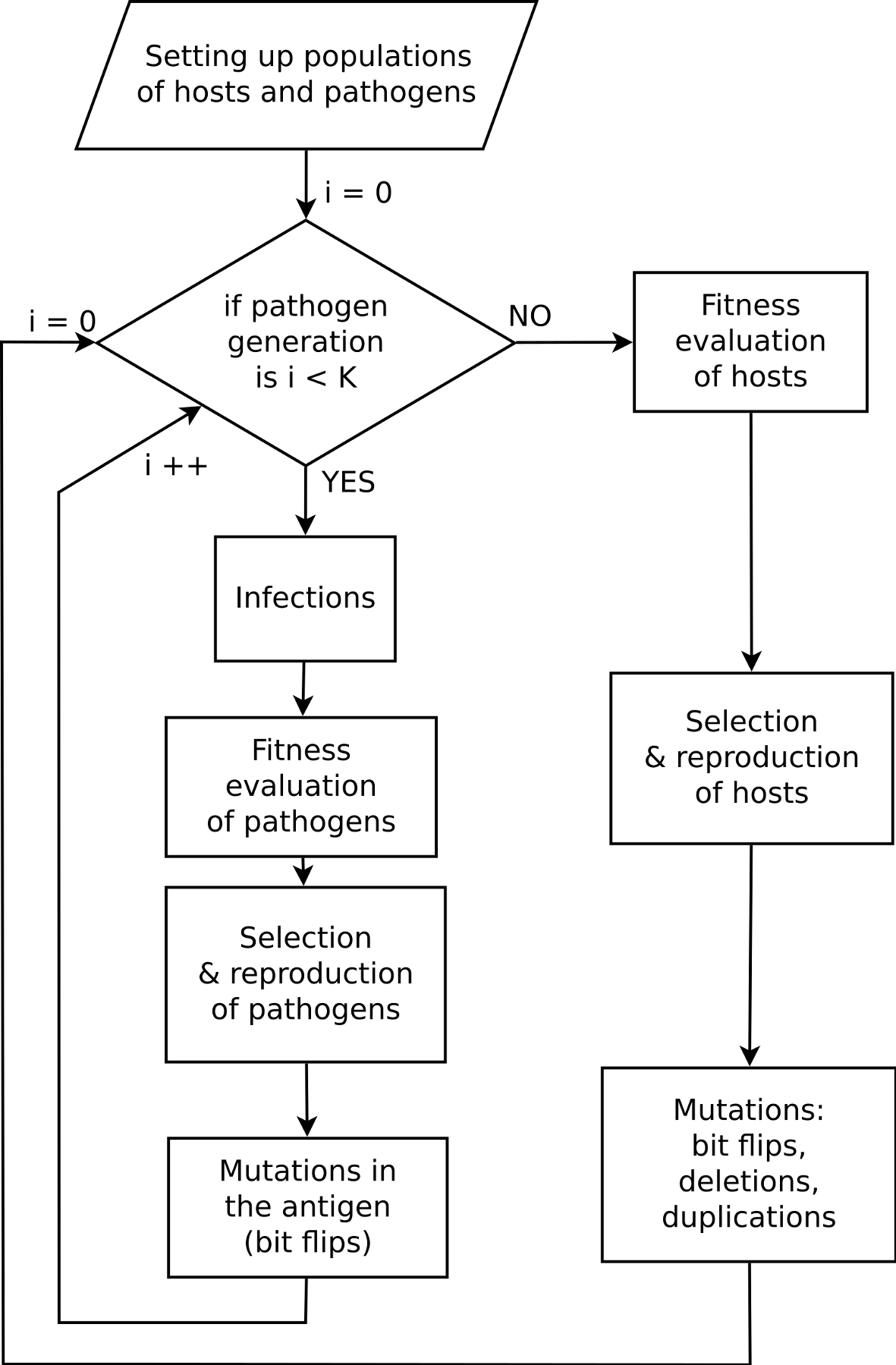

Supplement: S2 Fig — The inner loop represents 10 pathogen generations during one host generation (the outer loop). (TIF) [file pcbi.1007015.s002.tif]

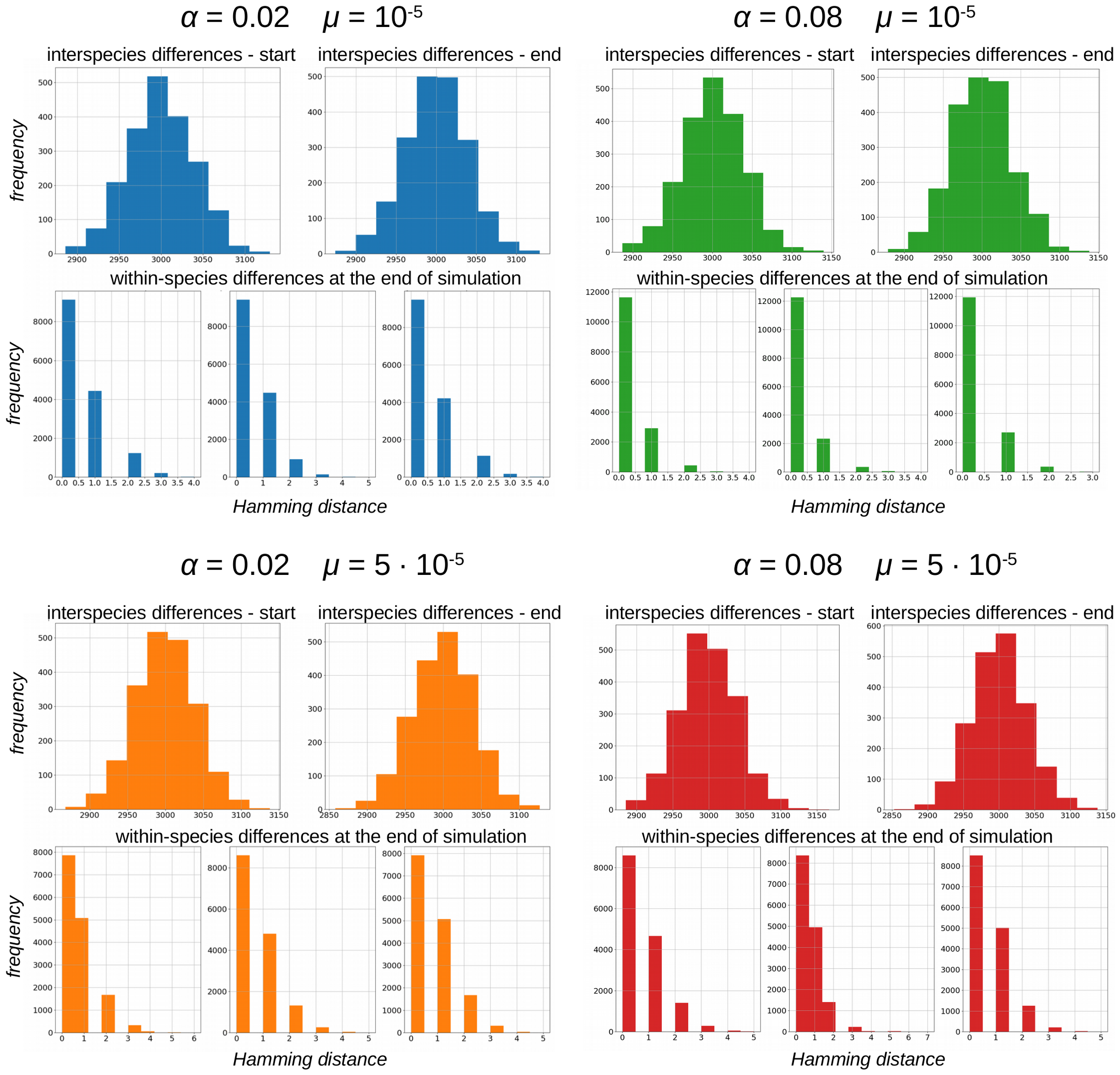

Supplement: S3 Fig — Presented are 4 runs with 64 pathogen species under two penalty parameters (α = 0.02; 0.08) and two pathogen mutation rates (μ A = 10–5; 5 ∙ 10–5). We used Hamming distance, a measure of similarity where two bit-strings of length n will have 1⁄2 n similar bits if they were generated randomly. At initialisation, a species will consist of a single copy of the same antigen, but species differ from each other. (TIF) [file pcbi.1007015.s003.tif]

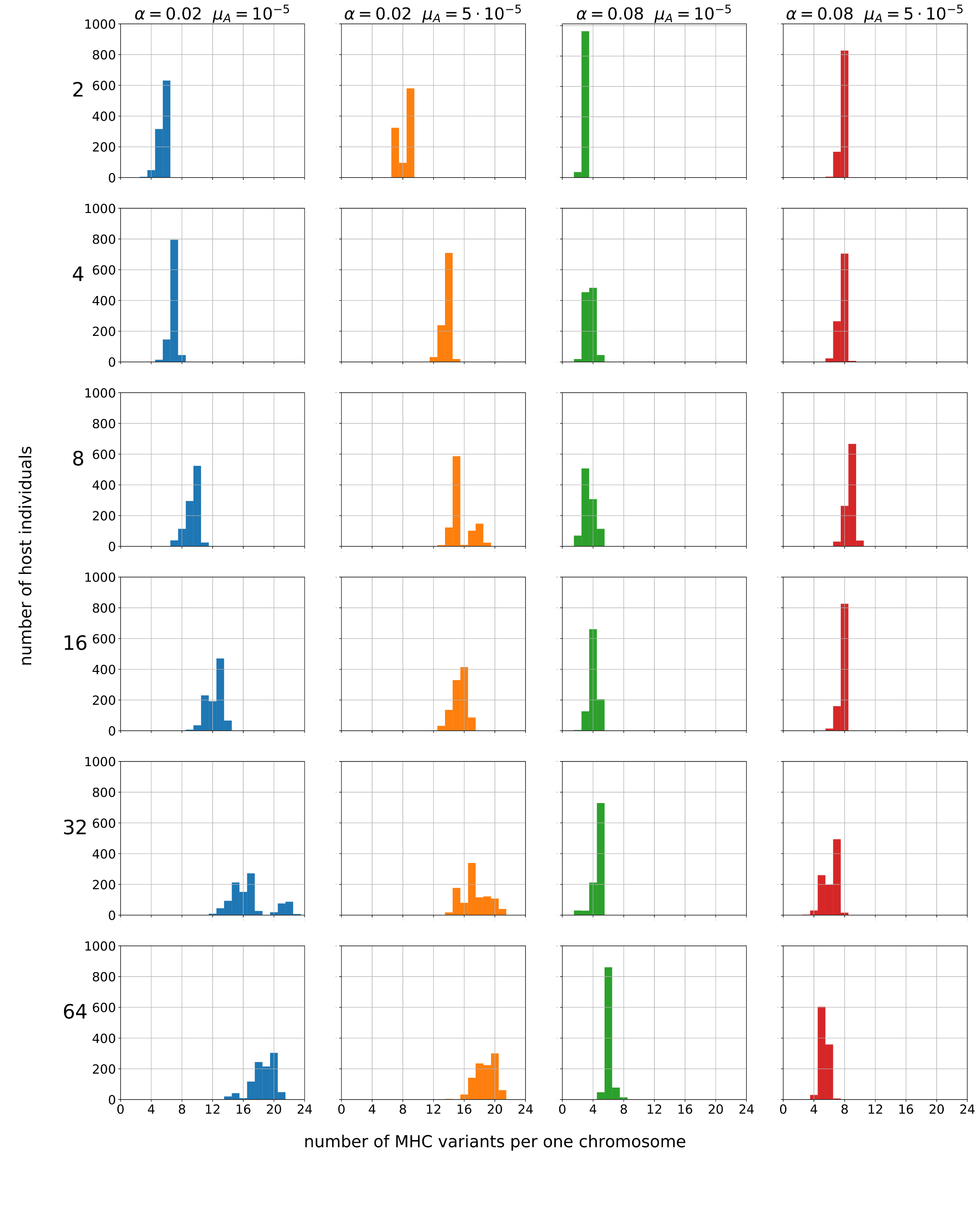

Supplement: S4 Fig — In columns are the two penalty parameters α = 0.02; 0.08 and two pathogen mutation rates μA = 10–5; 5 ∙ 10–5 (see descriptions above the figure). Rows contain runs with the same number of pathogen species (bold numbers on the right). Each panel represents one run that had its mean number of unique MHCs most similar to the mean calculated from all simulation of the same parametrization. (TIF) [file pcbi.1007015.s004.tif]

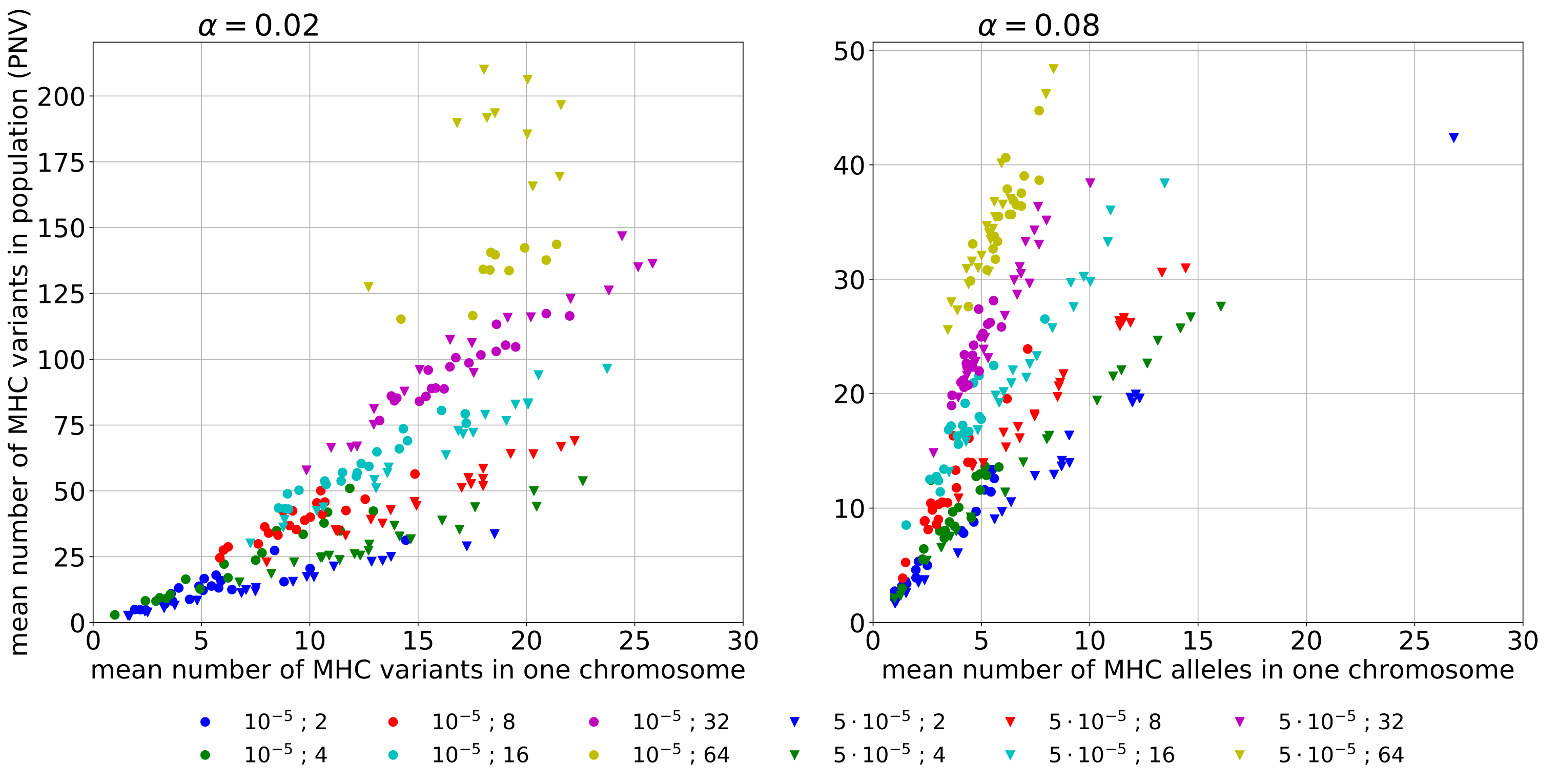

Supplement: S5 Fig — Note, the y-axes have different scales. On the legend dots indicate runs with pathogen mutation rates μA = 10–5, triangles μA = 5 ∙ 10–5. Colours correspond to the number of pathogen species in the simulation (see the legend beneath the panels). (TIF) [file pcbi.1007015.s005.tif]

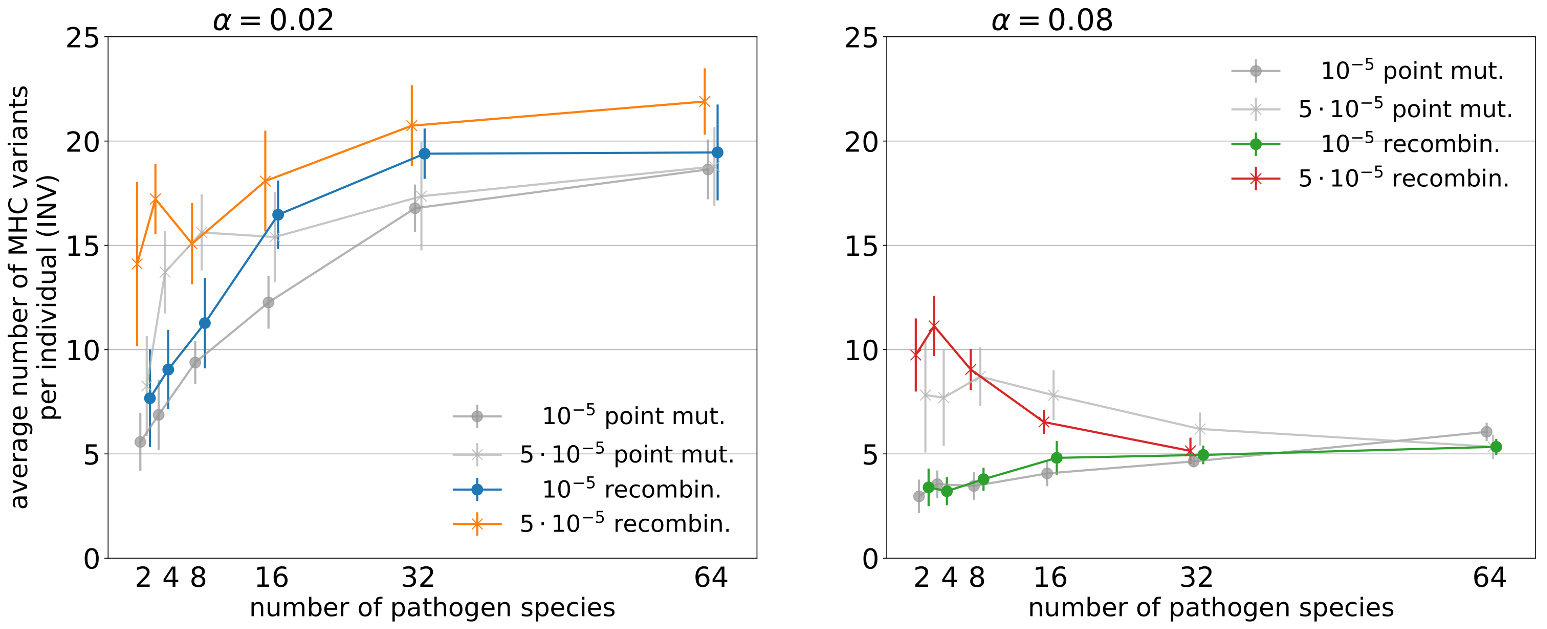

Supplement: S6 Fig — The points represent the mean of all the averaged values of simulations in a given parameter set with the 95% CI of the mean (bars). (TIF) [file pcbi.1007015.s006.tif]
